# Erectile Dysfunction and Cardiovascular Events in Diabetic Men: A Meta-analysis of Observational Studies

**DOI:** 10.1371/journal.pone.0043673

**Published:** 2012-09-04

**Authors:** Tomohide Yamada, Kazuo Hara, Hitomi Umematsu, Ryo Suzuki, Takashi Kadowaki

**Affiliations:** Department of Diabetes and Metabolic Diseases, Graduate School of Medicine, University of Tokyo, Tokyo, Japan; University of Tor Vergata, Italy

## Abstract

**Background:**

Several studies have shown that erectile dysfunction (ED) influences the risk of cardiovascular events (CV events). However, a meta-analysis of the overall risk of CV events associated with ED in patients with diabetes has not been performed.

**Methodology/Principal Findings:**

We searched MEDLINE and the Cochrane Library for pertinent articles (including references) published between 1951 and April 22, 2012. English language reports of original observational cohort studies and cross-sectional studies were included. Pooled effect estimates were obtained by random effects meta-analysis.

A total of 3,791 CV events were reported in 3 cohort studies and 9 cross-sectional studies (covering 22,586 subjects). Across the cohort studies, the overall odds ratio (OR) of diabetic men with ED versus those without ED was 1.74 (95% confidence interval [CI]: 1.34–2.27; P<0.001) for CV events and 1.72 (95% CI: 1.5–1.98; P<0.001) for coronary heart disease (CHD). The funnel plot, Begg's test, and Egger's test did not show evidence of publication bias (all P>0.05). Moreover, meta-regression analysis found no relationship between the method used to assess ED (questionnaire or interview), mean age, mean hemoglobin A_1c_, mean body mass index, or mean duration of diabetes and the risk of CV events or CHD. In the cross-sectional studies, the OR of diabetic men with ED versus those without ED was 3.39 (95% CI: 2.58–4.44; P<0.001) for CV events (N = 9), 3.43 (95% CI: 2.46–4.77; P<0.001) for CHD (N = 7), and 2.63 (95% CI: 1.41–4.91; P = 0.002) for peripheral vascular disease (N = 5).

**Conclusion/Significance:**

ED was associated with an increased risk of CV events in diabetic patients. Prevention and early detection of cardiovascular disease are important in the management of diabetes, especially in view of the rapid increase in its prevalence.

## Introduction

Cardiovascular disease (CVD) is the main cause of death in patients with diabetes, so its early detection is extremely important [1]. Erectile dysfunction (ED) is defined as the inability to achieve and maintain an erection for satisfactory sexual performance [2], and men with diabetes have a higher prevalence of ED compared with the general population [3]. Studies performed in various populations have found a frequency of ED ranging from 20% to 90%, depending on the method of assessment [4]–[6]. It has also been reported that the prevalence of ED increases with age and with the duration and severity of diabetes [7]. Several recent studies have shown that ED is associated with the risk of cardiovascular events (CV events) [8], [9], and have generally found a positive association, although its magnitude has varied between studies. In addition, two previous meta-analyses [10], [11] identified a statistically significant relation between ED and cardiovascular risk. However, a meta-analysis of the overall risk of CV events associated with ED in patients with diabetes, whose CVD risk is far higher than that of persons without diabetes, has not yet been performed. Clarifying the relationship between ED and CV events may facilitate the early detection of high-risk diabetic patients. Accordingly, we investigated the association of ED with CV events in men with diabetes by performing a meta-analysis.

## Methods

### Searches

The Medline and Cochrane Library electronic databases (from 1951 until April 22, 2012) were searched using the medical subject headings (MeSH) “Erectile Dysfunction”, “Diabetes”, and “Cardiovascular Disease” to identify observational studies that tested the association between ED and the risk of CV events, coronary heart disease, peripheral vascular disease, or stroke in diabetic men. The reference lists of pertinent articles were also reviewed.

### Selection

We performed initial screening of study titles or abstracts, while the second screening was based on full-text review. Cohort studies, case-control studies, and cross-sectional studies evaluating the risk of CV events in relation to ED were considered eligible for inclusion if the following criteria were met: 1) full-text report published in English; 2) reporting of event numbers in each exposure category; 3) reporting about the presence/absence of ED; and 4) reporting of CV events. If more than one study covered the same cohort, only the report containing the most comprehensive information on that population was included to avoid analysis of overlapping populations.

### Definition of CV events

CV events were defined according to terms in the history (including death from cardiovascular disease) and/or were classified as coronary heart disease (CHD). CHD included coronary artery disease, myocardial infarction, angina pectoris, cardiomyopathy, and other types of ischemic heart disease. Peripheral vascular disease (PVD) included peripheral artery disease, foot ulcers, and amputation of the lower limb.

### Assessment of validity

To ascertain the validity of the eligible studies, the quality of each report was appraised with reference to the STROBE statement [12].

### Data extraction

Two independent investigators (T.Y. and H.U.) reviewed each report to determine its eligibility and then extracted and tabulated all of the relevant data. Disagreement was resolved by consensus between the two authors. The following information was obtained from each article: first author, year of publication, type of diabetes, country, method of assessing ED, outcomes, follow-up period, total number of patients, age, hemoglobin A_1c_ (HbA_1c_), body mass index (BMI), duration of diabetes, and variables used for adjustment of analyses. Numerical data reported in the articles were used, and study authors were contacted if necessary to obtain further details. When available, adjusted relative risk estimates and the corresponding 95% confidence intervals (CIs) were extracted and used in the adjusted meta-analysis.

### Quantitative synthesis of data

A pooled odds ratio (OR) was calculated to evaluate the association between ED and CV events, CHD, or PVD across the studies by DerSimonian-Laird random effects meta-analysis. The equivalence of ORs between cohort studies and cross-sectional studies was assessed by z-statistic tests. Meta-regression analyses were performed to explore sources of heterogeneity. Variables such as the method used for assessment of ED (International Index of Erectile Function (IIEF) questionnaire or interview), mean age, mean HbA_1c_, mean BMI, and mean duration of diabetes were examined to detect any significant influence on the risk of CV events and CHD. Moreover, pooled ORs adjusted for possible confounders and their 95% CIs were calculated for the risk of CV events in the cohort studies by the random-effect model weighted with inverse variance. The Cochrane χ2 test and the I-squared test were used to evaluate heterogeneity among studies, with a threshold value of p = 0.10 being considered significant [13]. Publication bias was evaluated by creating a funnel plot of each study's effect size versus the SE. Funnel plot asymmetry was assessed by Begg's test and Egger's test. Then trim-and-fill computation was used to estimate the influence of publication bias [14]. All statistical analyses were performed with Stata 11.0 software (StataCorp, College Station, TX). Results are expressed as the mean with 95% CI, unless otherwise indicated. Except for tests of heterogeneity, a P value of less than 0.05 was considered significant. All procedures were performed in accordance with the guideline for the meta-analysis of observational studies in epidemiology [15] and the PRISMA statement [16].

## Results

### Search results

**Figure 1:**
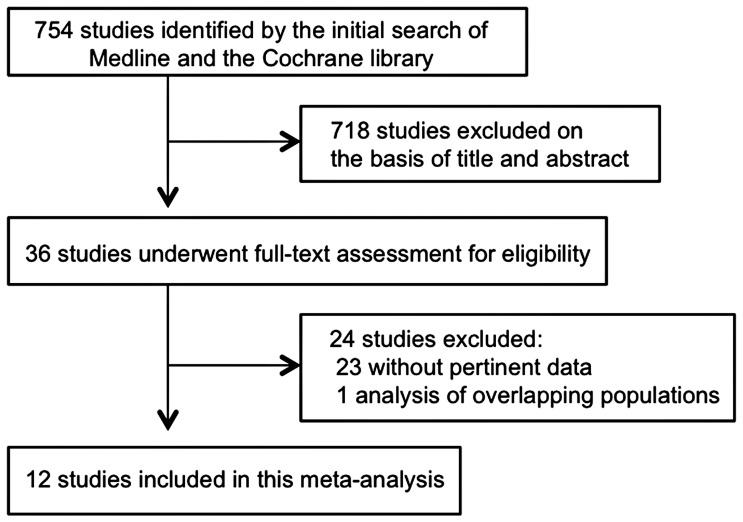
Flow diagram of study selection.

**Figure 1** shows a flow diagram of study selection. We identified a total of 754 citations by the two database searches. Of these citations, 718 were excluded by reviewing the title and abstract, leaving 36 studies for further evaluation. Twenty-four of these 36 studies were excluded after full-text evaluation. Most of the excluded studies did not contain pertinent data, while 1 was excluded because of multiple publications. One report [17] was included after we obtained the event numbers used in the original calculations from its author. A total of 12 studies [7], [17]–[27] that covered 22,586 patients eventually fulfilled our inclusion criteria and were used in this meta-analysis.

### Study characteristics

The 12 investigations [7], [17]–[27] included in this meta-analysis consisted of 3 cohort studies [17]–[19] and 9 cross-sectional studies [7], [20]–[27]. **Table 1** shows their characteristics. There was moderate heterogeneity of study design, the type of diabetes, and the method used for assessment of ED. The studies were published between 1996 and 2011. Eight studies [17]–[19], [22], [24]–[27] only analyzed type 2 diabetic patients, 1 [20] was only performed on type 1 diabetic patients, and 3 [7], [21], [23] covered both types of diabetes. Six studies [19], [21], [22], [24], [26], [27] were conducted in Europe, 2 [18], [25] in Asia, 2 [7], [23] in the Middle East, 1 [20] in the USA, and 1 [17] in multiple countries. All 12 studies reported CV events, while 10 [7], [17]–[19], [21], [23]–[27] reported on CHD, 6 [19], [20], [22], [23], [25], [27] reported on PVD. For the 3 cohort studies [17]–[19], the mean follow-up period ranged from 3.9 to 5.0 years. The size of the study population ranged from 154 to 9,752 patients (mean: 1,882 patients). The age, HbA_1c_, BMI, and duration of diabetes were largely in the range between 50–60 years, 7.0–8.0%, 25–28 kg/m^2^, and 5.0–10.0 years, respectively. The definition and method of assessing ED varied across the studies, with 7 [7], [19], [23]–[27] studies being based on the IIEF questionnaire and 5 [17], [18], [20]–[22] studies using interview (e.g., asking patients whether they had ED).

**Table 1:** Summary of studies evaluating the association between ED and CV events in diabetic men.

First author, year	Type of diabetes	Country	Assessment of ED	Outcomes	Follow-up (yrs)	Number of all patients	Age (yrs)	HbA_1c_ (%)	BMI (kg/m^2^)	Duration of diabetes (yrs)
Cohort studies										
Ma, 2008^a^ [18]	Type 2	Hong Kong (China)	Interview	CV events, CHD	4.0	2306	54.2	7.8	25.0	5.9
Gazzaruso, 2008^b^ [19]	Type 2	Italy	IIEF-5 questionnaire	CV events, CHD, PVD	3.9	291	54.8	7.3	27.5	8.2
Batty, 2010^c^ [17]	Type 2	Multiple countries	Interview	CV events, CHD	5.0	6304	65.9	7.5	28.0	8.0
Cross-sectional studies										
Klein, 1996^d^ [20]	Type 1	USA	Interview	CV events, PVD	-	359	37.6	10.0	25.7	22.5
Fedele, 2000^e^ [21]	Type 1 and Type 2	Italy	Interview	CV events, CHD	-	9752	20–69	NA	NA	NA
Kalter-Leibovici, 2005^f^ [7]	Undefined	Israel	IIEF-15 questionnaire	CV events, CHD	-	1040	57.0	7.7	28.5	8.0
Berardis, 2005^g^ [22]	Type 2	Italy	Interview	CV events, PVD	-	1264	61.3	7.0	27.4	10.1
Shiri, 2006^h^ [23]	Undefined	Iran	IIEF-5 questionnaire	CV events, CHD, PVD	-	312	55.2	NA	NA	7.6
Gazzaruso, 2006^i^ [24]	Type 2	Italy	IIEF-5 questionnaire	CV events, CHD	-	198	57.8	7.5	26.3	7.5
Yu, 2010^j^ [25]	Type 2	Hong Kong (China)	IIEF-5 questionnaire	CV events, CHD, PVD	-	313	56.1	7.6	26.0	9.3
Gazzaruso, 2011^k^ [26]	Type 2	Italy	IIEF-5 questionnaire	CV events, CHD	-	293	56.6	7.8	27.0	0
Malpartida, 2011^l^ [27]	Type 2	Spain	IIEF-15 questionnaire	CV events, CHD, PVD	-	154	55.9	6.7	30.2	5

BMI, body mass index (calculated as weight in kilograms divided by height in meters squared); CV events, cardiovascular events; CHD, coronary heart disease; PVD, peripheral vascular disease; IIEF, International Index of Erectile Function;

a Adjusted for age, duration of diabetes, albuminuria, and use of antihypertensive medications.

b Adjusted for age, duration of diabetes, hypertension, family history of CHD, smoking, microalbuminuria, HbA_1c_, BMI, total cholesterol, triglycerides, low-density lipoprotein cholesterol, high-density lipoprotein cholesterol, and autonomic dysfunction.

c Adjusted for age, BMI, use of metformin or beta-blockers, history of macrovascular or microvascular disease, duration of diabetes, smoking, alcohol intake, physical activity, HbA_1c_, creatinine, total cholesterol, high-density lipoprotein cholesterol, resting heart rate, blood pressure, and education.

d Adjusted for age, duration of diabetes, and HbA_1c_.

e Adjusted for age and duration of diabetes.

f Adjusted for age, diabetes duration, HbA_1c_, microvascular disease, diuretic therapy, work-related and leisure-time physical activity, and alcohol consumption.

g Not adjusted.

h Adjusted for age, education, type and duration of diabetes, pulmonary disease, depression, fruit intake, smoking, drugs and substance abuse, microalbuminuria, and HbA_1c_.

i Adjusted for age, duration of diabetes, hypertension, family history of coronary artery disease, smoking, microalbuminuria, HbA_lc_, BMI, cholesterol, triglycerides, LDL, and HDL.

j Adjusted for age, duration of diabetes, HbA_1c_, insulin therapy, hypertension, dyslipidemia, log albumin/creatinine ratio, retinopathy, chronic kidney disease, and cerebrovascular disease.

k Adjusted for HbA_1c_, BMI, cholesterol, triglycerides, LDL, HDL, hypertension, dyslipidemia, family history of coronary artery disease, smoking, microalbuminuria/macroalbuminuria, pharmacologic treatment, and autonomic dysfunction.

l Adjusted for age and duration of diabetes.

Most of the studies at least used the age and duration of diabetes for adjustment (similar to the cohort studies), but the number of variables differed significantly among the studies.

Only 2 reports on cross-sectional studies [24], [25] explicitly mentioned the limitations inherent in a cross-sectional design (it cannot explain causality), the possible biases of the study, or the influence of confounders on the results. The other reports on cross-sectional studies lacked this type of statement. In contrast, confounders were satisfactorily adjusted and limitations were fully described in the reports on the 3 cohort studies [17]–[19].

### Quantitative data synthesis (meta-analysis)

A total of 3,791 CV events were reported in the 3 cohort studies and 9 cross-sectional studies covering a total of 22,586 subjects, including 2,229 events in 9,480 subjects with ED and 1,562 events in 13,106 subjects without ED. None of the studies revealed a lower risk of CV events in patients with ED.

**Figure 2:**
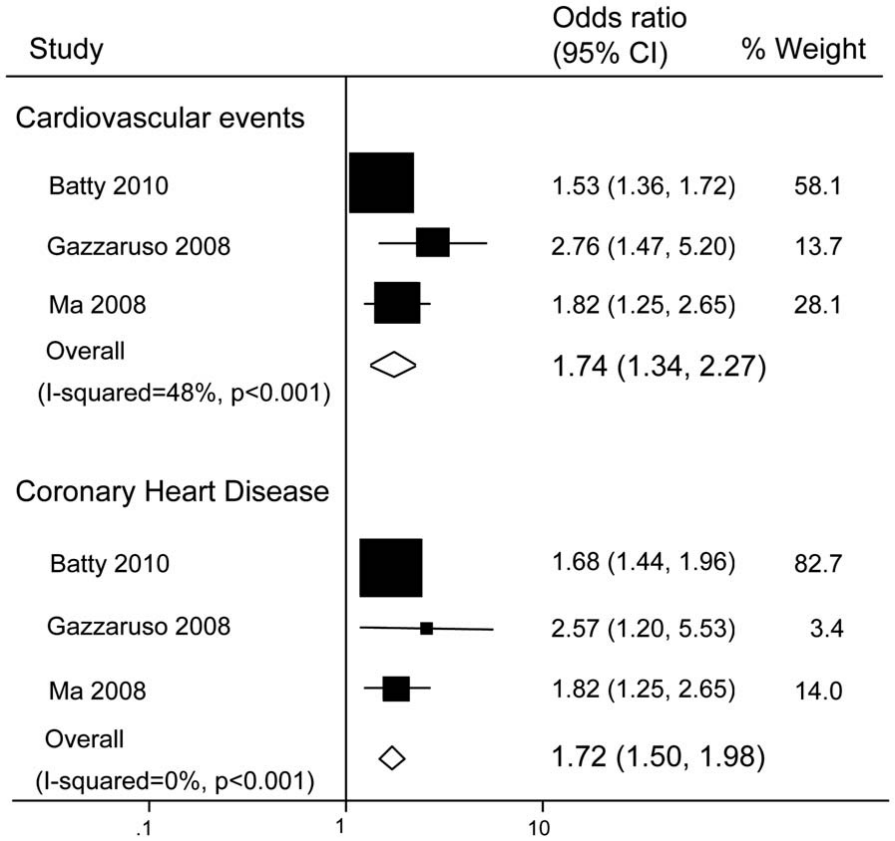
Pooled odds ratios for the risk of cardiovascular events and coronary heart disease in diabetic men (cohort studies).

Across the cohort studies, the overall odds ratio (OR) for diabetic men with ED versus those without ED was 1.74 (95% confidence interval [CI]: 1.34–2.27; P<0.001; P for heterogeneity = 0.15; I-squared = 48%) for CV events and 1.72 (95% CI: 1.5–1.98; P<0.001; P for heterogeneity = 0.54; I-squared 0%) for CHD (**Fig. 2**). Substantial heterogeneity was not observed.

**Figure 3:**
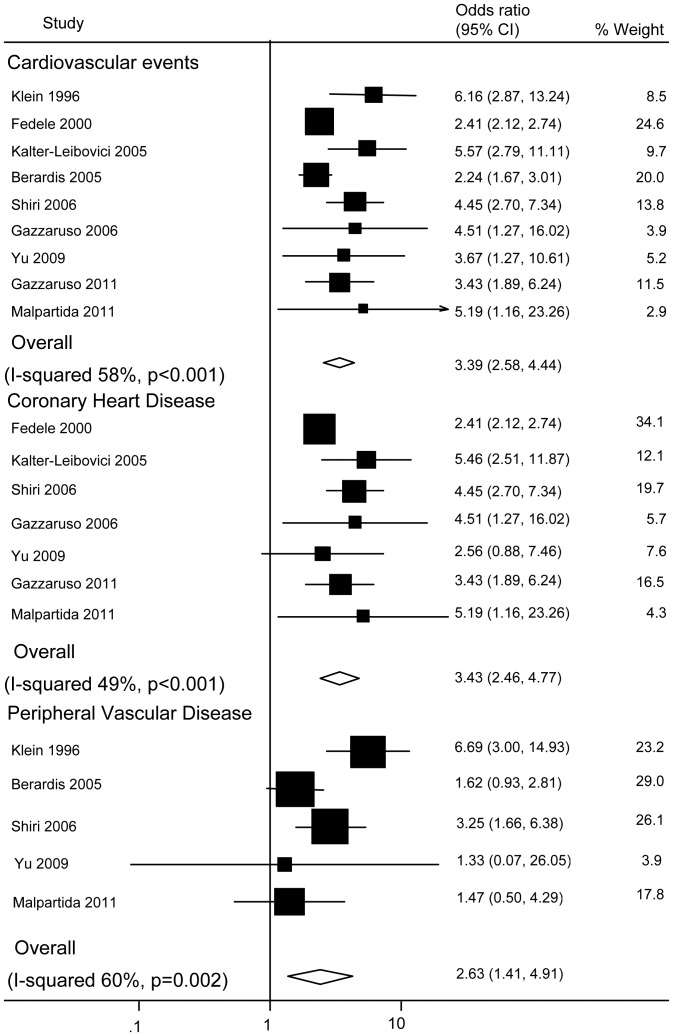
Pooled odds ratios for the risk of cardiovascular events, coronary heart disease, and peripheral vascular disease in diabetic men (cross-sectional studies).

Across the cross-sectional studies, the OR for diabetic men with ED versus those without ED was 3.39 (95% CI: 2.58–4.44; P<0.001; P for heterogeneity = 0.014; I-squared = 58%) for CV events (N = 9), 3.43 (95% CI: 2.46–4.77; P<0.001; P for heterogeneity = 0.07; I-squared = 49%) for CHD (N = 7), and 2.63 (95% CI: 1.41–4.91; P = 0.002; P for heterogeneity = 0.04; I-squared = 60%) for PVD (N = 5) (**Fig. 3**).

The difference of ORs for CV events and CHD between the cohort studies and cross-sectional studies was statistically significant (both p<0.01) according to the z-statistics test.

### Meta-regression analysis

We performed meta-regression analysis separately for the 3 cohort studies and the 9 cross-sectional studies.

Since we only found 3 cohort studies, we were not able to perform multivariate meta-regression analysis. Instead, univariate meta-regression analysis was done using the variables of (1) age, (2) HbA_1c_, (3) BMI, (4) duration of diabetes, and (5) method of assessing ED (IIEF questionnaire or interview). As a result, none of these covariates showed a significant relation with the risk of CV events (BMI, P = 0.83; age, P = 0.28; HbA_1c_, P = 0.81; duration of diabetes, P = 0.98; and assessment of ED, P = 0.38) or with the risk of CHD (BMI, P = 0.73; age, P = 0.52; HbA_1c_, P = 0.91; duration of diabetes, P = 0.88; and assessment of ED, P = 0.49).

Next, we conducted a multivariate meta-regression analysis of CV event risk using data from the cross-sectional studies. Again, none of the above-mentioned covariates (1) to (5) had a significant influence on the risk of CV events (BMI, P = 0.48; age, P = 0.96; HbA_1c_, P = 0.81; duration of diabetes, P = 0.88; and assessment of ED, P = 0.92). Moreover, univariate meta-regression analysis revealed no significant variables (BMI, P = 0.54; age, P = 0.15; HbA_1c_, P = 0.27; duration of diabetes, P = 0.35; and assessment of ED, P = 0.8).

Regarding the risk of CHD events, 5 studies used the three variables of age, HbA_1c_, and duration of diabetes for adjustment. Accordingly, we conducted a multivariate meta-regression analysis of those 5 studies using these 3 variables. However, none of the 3 variables showed a significant relation with CHD (age, P = 0.58; HbA_1c_, P = 0.99; and duration of diabetes, P = 0.95). Univariate meta-regression analysis also found no significant variables (BMI, P = 0.11; age, P = 0.33; HbA_1c_, P = 0.77; duration of diabetes, P = 0.91; and assessment of ED, P = 0.95).

### Adjusted meta-analysis

The relative risk (RR) of CV events in the 3 cohort studies was adjusted for potential confounders. Meta-analysis performed with these adjusted RRs showed that the presence of ED still predicted CV events, with an RR of 1.57 (95% CI: 1.04–2.36; P = 0.03; P for heterogeneity<0.001; I-squared = 91%)

### Publication bias

The funnel plot did not show an asymmetric pattern, and both Begg's test and Egger's test revealed no significant publication bias (Begg's test, P = 0.12 and Egger's test, P = 0.10 for CV events; Begg's test, P = 0.12 and Egger's test, P = 0.18 for CHD).

Trim-and-fill computation showed that the identified bias did not interfere with interpretation of the results (OR: 1.53 (95% CI: 1.21–1.94), P<0.001 for CV events; OR; 1.68 (95% CI: 1.48–1.91), P<0.001 for CHD).

## Discussion

This meta-analysis of 12 studies from around the world demonstrated that ED is associated with a substantial increase in the risk of CV events, CHD, and PVD in diabetic men. Our findings have implications for the management of diabetes, especially in view of the rapid increase in the prevalence of this disease.

Diabetic men with ED suffer a significant decline in quality-of-life measures [22], but their symptoms may remain unnoticed because many physicians do not inquire about sexual health. It has been reported that the majority of men with diabetes and ED have never been asked about sexual function by their physicians, and therefore do not receive treatment for ED [3]. Our findings suggest that, as already reported [28], ED could be a marker of silent CVD and that silent CVD should be excluded before starting to treat ED.

There have been two previous meta-analyses of the relationship between ED and CVD [10], [11], but they were not limited to diabetic patients. The combined RR from both meta-analyses was about 1.5 (1.48 [10] and 1.47 [11]) for CVD. In the present meta-analysis, the estimated RR was a high 1.74. Therefore, it seems that the risk of CVD in men with type 2 diabetes and ED is equivalent or higher than that for non-diabetic men with ED.

### Mechanism of the relation between ED and CVD

A normal erection is achieved by an increase of parasympathetic activity and a reduction of sympathetic activity [29]. Several mechanisms to explain the association between ED and CVD have been postulated. One is the “artery size hypothesis” [30]. Atherosclerosis affects all major vascular beds to a similar extent, but the penile arteries have a smaller diameter than the coronary arteries (1–2 mm vs. 3–4 mm) and thus are affected earlier by accumulation of atherosclerotic plaque, so that the onset of ED may precede vascular events in the heart. Another possible explanation is that endothelial dysfunction may be a shared etiologic factor for both diseases [31]. Endothelial dysfunction without atherosclerotic narrowing of the penile arteries is more likely to cause ED than it would be to cause angina if the coronary arteries were similarly affected [32]. There may also be smooth muscle dysfunction as well as endothelial dysfunction in patients with ED, which could occur before onset of systemic vascular disease [33]. Moreover, ED is a major clinical manifestation of diabetic autonomic neuropathy. Autonomic neuropathy impairs cholinergic activation of the erectile process and interferes with autonomic pelvic nerve stimulation and/or corporal nerve release of endogenous neurotransmitters [34].

It should be noted that many of the patients analyzed in our study had type 2 diabetes. There may be differences between type 1 and type 2 diabetes with regard to the relationship of ED and CVD, such as a difference in the age of onset or the prevalence of concomitant medical conditions that are also risk factors for cardiovascular disease, including hypertension and dyslipidemia.

### CVD screening in asymptomatic diabetic patients

Recently, there have been many reports about the usefulness of CVD screening in asymptomatic diabetic patients. Type 2 diabetes is associated with an elevated risk of coronary artery disease, but patients are often asymptomatic [35], and the usefulness of screening this population is yet to be elucidated. It has been reported that the incidence of coronary artery disease was not significantly reduced when screening by myocardial scintigrapy was conducted in asymptomatic diabetic patients [36], and it was also reported that coronary CT screening of asymptomatic patients without a history of coronary artery disease was not effective for preventing major cardiovascular events [37].

On the other hand, taking a detailed history was reported to be effective for predicting cardiovascular events in high-risk outpatients [38], while systematic assessment of the family history is useful for evaluating cardiovascular risk [39]. Therefore, regardless of the method employed, screening can be recommended for patients who are considered to have a high risk of cardiovascular events based on their history or clinical findings. The method employed should be practicable in terms of its advantages/disadvantages and cost performance, and patients should be evaluated for eligibility before screening. We consider that taking a history of sexual function, hypertension, and dyslipidemia, as well as a family history of CVD, is helpful for assessing the risk of coronary heart disease.

### Limitations

The present analysis had several limitations. First, there is a possibility that relevant research papers were missed (e.g., those not written in English), resulting in selection bias. Second, substantial heterogeneity was observed among the studies, which suggested that the different study categories (cohort or cross-sectional) contributed to this heterogeneity to some extent. Although substantial heterogeneity led to a wide range of plausible risk estimates, we found no evidence to suggest that ED is associated with a lower risk of CV events. The present findings may also reflect the differing epidemiological characteristics of the patient populations included in our meta-analysis. Although CVD confounders did not explain the heterogeneity according to meta-regression analysis, it must be remembered that it is impossible to avoid the influence of measured (and unmeasured) confounders (such as age, obesity, dyslipidemia, alcohol, exercise, endocrine disorders, and ejaculatory dysfunction) in observational studies.

Moreover, although our meta-analysis of the cohort studies and cross-sectional studies revealed a significant difference of the ORs for CV events and CHD, this difference may have been influenced by the limitations inherent in cross-sectional studies and the biases of the studies we analyzed.

Third, there was limited information about the use of medications such as antidepressants, beta-blockers, diuretics, phosphodiesterase inhibitors, testosterone, and antihypertensive agents that may have contributed to ED. Fourth, the method used for assessment of ED varied between studies. The IIEF questionnaire has been adopted as the gold standard when assessing the efficacy of treatment for ED [40]. It provides information about the severity of ED and allows dose-response effects to be examined. Conversely, other methods such as interviewing the subject have a higher likelihood of misclassification bias that could lead to underestimation of the strength of the association, since it is often considered shameful to admit to the existence of ED. However, meta-regression analysis did not identify a significant difference between studies based on the IIEF questionnaire and those based on interview.

Even with these limitations, observational studies can provide useful evidence regarding the potential influence of ED on CV events and the overall pooled estimates were robust. Moreover, there are both clinical and biochemical evidence supporting a relation between ED and CV events, as discussed above. Our findings should prompt physicians to ask diabetic men about ED. In addition, the relationship between ED and CVD should be investigated by further studies, including well-designed and carefully controlled cohort studies, in order to confirm whether identification of ED facilitates the early detection of diabetic men with a high risk of CVD.

### Conclusions

The presence of ED was associated with an increased risk of CV events in diabetic men. Prevention and early detection of CVD are important in the management of diabetes, especially in view of the rapid increase in its prevalence.

## Supporting Information

Checklist S1 **PRISMA Checklist.**
(DOC)
